# Effects of Several Preharvest Canopy Applications on Yield and Quality of Table Grapes (*Vitis vinifera* L.) *Cv*. Crimson Seedless

**DOI:** 10.3390/plants10050906

**Published:** 2021-04-30

**Authors:** Despoina G. Petoumenou, Vasileios-Emmanouil Patris

**Affiliations:** Laboratory of Viticulture, Department of Agriculture Crop Production and Rural Environment, University of Thessaly, 38446 Volos, Greece; vpatris@uth.gr

**Keywords:** biostimulants, *Vitis vinifera*, anthocyanins, seaweed extract, *Ecklonia maxima*, *Ascophyllum nodosum*, *Saccharomyces cerevisiae*, Ethrel^®^ Top, Sunred^®^, viticulture

## Abstract

Modern viticultural areas are being confronted with the negative impacts of global warming on yield and fruit composition, with especially adverse effects on anthocyanin synthesis. Novel and sustainable tools, such as biostimulants, may represent a viable alternative to traditional cultural practices, thus promoting eco-friendly strategies to enhance the yield, fruit quality and abiotic stress tolerance of grapevines. ‘Crimson Seedless’ is a late-season red table grape variety, and due to climatic warming, its berries are frequently failing to acquire the commercially acceptable red color. Canopy applications of different biostimulants, namely, Kelpak^®^, Sunred^®^, Cytolan^®^, LalVigne™ Mature as well as Ethrel^®^ Top, were tested on grapevine *cv*. Crimson Seedless grown under semi-arid Mediterranean conditions in order to evaluate their effects on yield and fruit quality. Some of the products were sprayed in canopies at labeled doses, and some were applied at doses reported in other studies. For the control treatment, canopies were sprayed with water. Sampling started at veraison and was repeated at 10-day intervals to measure the evolution of berry weight, length and diameter, as well as the total soluble solids and titratable acidity of the juice. The grapes were harvested when the berries of one of the treatments attained the commercially acceptable color. The greatest improvements in the red berry color were achieved with Sunred^®^ (at a dose of 4 L ha^−1^) and Ethrel^®^ Top (250 ppm plus glycerol at 1%), each applied at veraison and 10 days later. The different applications had varying effects on productivity and qualitative parameters. Only Sunred^®^ improved the accumulation of anthocyanin and the overall acceptability of table grapes by consumers. The obtained results clearly demonstrate that applying Sunred^®^ can improve the yield and qualitative parameters of the red table grape variety ‘Crimson Seedless’, indicating that this biostimulant could be a viable alternative to the most widely used plant growth regulator, ethephon.

## 1. Introduction

One of the contemporary challenges of table grape production is continuing to satisfy consumer standards for high and consistent fruit quality, regardless of seasonal variations in yield and grape composition caused by changing environmental conditions [1].

Several environmental factors, such as temperature, solar radiation and precipitation, can potentially exert a wide range of effects on vine production and berry quality [2]. In particular, temperature has been associated with inadequate grape skin color [3]. High summer temperatures have been reported to influence anthocyanin synthesis [4,5]. Moreover, a narrow temperature range between day and night in the summer [6] or elevated night temperatures [7,8] are believed to exacerbate this situation. Indeed, the higher summer temperatures that occur during the most sensitive phenological stages, which is 1–3 weeks after the beginning of veraison [9], can significantly modify the content and composition of anthocyanins in grape berries by affecting the gene expression involved in anthocyanin biosynthesis [10]. A direct consequence is non-optimal grape quality because of the uneven color of some bunches, which forces grape growers to perform numerous harvests, thus increasing production costs. Therefore, a range of strategies aimed at correcting and/or preventing these issues have been introduced, including the application of growth regulators such as ethephon and/or abscisic acid during berry ripening. These substances are widely used on Crimson Seedless, a late-season red table grape cultivar [11,12]. The exogenous application of ethylene stimulates the expression of genes related to anthocyanin biosynthesis [13], and thus, it is used for improving berry skin color and accelerating maturation in grapevines [14].

Biostimulants represent possible viable alternatives to growth regulators for table grape production. A plant biostimulant is defined as any substance or microorganism that, regardless of its nutrient elements, can improve nutrition efficiency, crop quality characteristics and/or abiotic stress resistance when applied to plants [15]. Seaweed extracts are organic and biodegradable substances and are considered an important source of nutrition in sustainable agriculture [16]. There are numerous seaweed species, with more than 10,000 red, brown and green seaweed species [17]. Some brown seaweed species are used more frequently, such as *Ascophyllum nodosum* (L.) Le Jol., *Ecklonia maxima*, *Macrocystis pyrifera* and *Durvillea potatorum* [17]. According to Metting et al. [18], plants that are treated with seaweed extracts had increased crop yield, nutrition uptake, seed germination and postharvest shelf-life, and they were also resistant to stress conditions, such as high temperature or frost, and less susceptible to insect attacks and fungal infections. Foliar applications of *Ascophyllum nodosum* seaweed have been reported to have a wide range of beneficial effects on plants. This species has been demonstrated to increase the germination of bean seeds [19], promote the concentration of flavonoids and phenolics in cabbage [20] and increase the yield and curd diameter of cauliflower [21] and pepper plants [22]. *A. nodosum* has been reported to influence the growth, productivity and fruit quality of different varieties of grapes, such as ‘Flame Seedless’ [23], ‘Perlette’ [24], ‘Sangiovese’ [25] and ‘Trakya Ilkeren’ [26]. *Ecklonia maxima* is another species of seaweed extract with positive effects on plants after foliar applications [27,28]. It has been reported to improve the quality of ‘Merlot’ and ‘Cabernet Sauvignon’ grapes [29] and the firmness of ‘Crimson Seedless’ [30]. Inactivated yeasts, such as *Saccharomyces cervicisae* L., are another category of plant biostimulants; they have been reported to contain amino acids, vitamins and growth factors and enhance the uptake of various nutrients [31].

Since biostimulants are characterized by the beneficial properties mentioned above, they may be a viable alternative to the growth regulators used in table grape production. Therefore, the purpose of the present study was to investigate the impacts of several biostimulants, as well as those of ethephon, on the yield and quality of cv. ‘Crimson Seedless’ grapes, with a focus on enhancing the red color of the berries.

## 2. Materials and Methods

### 2.1. Vineyard Microclimate

The climate of the study site is Mediterranean, with mild wet winters and hot, dry summers. Environmental conditions such as precipitation (mm) and monthly maximum (T max) and minimum (T min) temperatures were logged by an automated weather station that was located near the vineyard and provided by the National Observatory of Athens with the supervision of the Municipality of Dion (Pieria, Greece). Same data of the ten previous years and for the same period were also gathered in order to compare them with the climate data in the experimental year.

### 2.2. Plant Material and Experimental Design

The experiment was conducted in the 2018/2019 production cycle in a commercial vineyard in north Greece (Karitsa, Pieria, 40°11′01.0″ N, 22°28′02.8″ E, 19 m a.s.l., loamy soil type, north–south row orientation). Twelve-year-old vines of *Vitis vinifera L. cv*. ‘Crimson Seedless’ grafted on 1103 Paulsen of similar vigor and health were used in the study. Vines were planted at 1.20 × 3.00 m between vines and rows, respectively, and trained to a lyre trellis system. The vines were cane-pruned in winter to ∼10 canes/vine; canes contained 10–12 buds each.

Cordons were trained 0.9 m aboveground. Three pairs of catch wires formed canopy walls of 1.2 m above the cordons, and vines were drip-irrigated at 3500–4000 m^3^/ha. Fertilization, pest control and cultural practices (berry thinning, leaf removal, shoot thinning and shoot trimming) were conducted according to local practices. Specifically, berry thinning was performed by applying 35 g of GA3 per ha at 30% caps off (E-L 21; modified E-L system, [32]), which was repeated after 5 days. For berry sizing, 55 g of GA3 per ha was applied at berry development (E-L 31–33). Basal leaves and leaves surrounding clusters were removed after the berry softening stage (E-L 35), taking care not to cause sunburns, and finally, shoot trimming was performed when the shoot tips exceeded the height of the top wire at ~15 cm.

A randomized complete block design was used, and the treatments were arranged in 12 blocks, represented by 12 rows of 12 vines per treatment; this design left one untreated row as a border between adjacent experimental units. Vines were assigned to the following treatments: (i) Kelpak^®^ treatment; (ii) Cytolan^®^ Concentrated Powder (Promisol S.A., Lleida, Spain) based on *Ascophyllum nodosum* (AN) extract (100% seaweed extract); (iii) LalVigne™ Mature (LM, Lallemand Inc., Montreal, QC, Canada), a natural product consisting of inactivated wine yeast (*Saccharomyces cerevisiae*) derivatives; (iv) Ethrel^®^ Top (Bayer S.A.S.—Bayer CropScience, Lyon, France) with 1% glycerin *v*/*v*; (v) Sunred^®^ (Biolchim, S.p.a., Medicina, Bologna, Italy) and (vi) water, which was applied to control vines. The products were applied at determined phenological stages and rates, as indicated in Table 1, which also reports their chemical compositions. All treatments were applied with a battery-powered backpack sprayer for a full canopy spray until runoff was observed.

### 2.3. Evolution of Berry Development and Grape Chemical Composition

At veraison, three samples of 50 representative berries were taken from each treatment. These samples were used to start monitoring the evolution of the berry weight, length and equatorial diameter. Sampling was performed until harvest at 10-day intervals, except for the 24th of September (44 Days After Veraison, DAV) and the 7th of October (57 DAV): these samples were collected after the 10-day interval due to rainfall on the scheduled days. The 50 berry samples were weighed with a balance (Kern & Sohn GmbH, Balingen, Germany) with ±0.1 precision, and the berry length and equatorial diameter were measured using a digital vernier caliper (Mitutoyo, Kawasaki, Japan) with 0.01 mm accuracy. After these measurements, the samples were crushed, and the below analyses were performed on their juice. Total soluble solids (TSS) were measured using a digital hand-held “pocket” refractometer PAL (Atago Co., Ltd., Tokyo, Japan) and expressed in °Brix at 20 °C. Titratable acidity (TA) was determined by titrating the grape juice with a 0.1 N sodium hydroxide (NaOH) solution in the presence of a bromothymol blue indicator. The results were expressed as the percentage of tartaric acid (%, g tartaric acid/100 mL juice). The maturity index was calculated as the TSS/TA ratio.

### 2.4. Harvest Data

At harvest, which was performed on the 7th of October (57 DAV) 2019, when the clusters of at least one of the treatments achieved the typical commercial red color, all the vines were handpicked carefully, and the yield per vine and total number of clusters per vine were recorded at the same time. Twenty representative clusters from each treatment were sampled, placed in a cooler and immediately brought to the laboratory, where cluster dimensions (weight, length and width), the number of berries per cluster and berry weight were measured, and the number of berries affected by bunch rot was visually determined. The bunch compactness index was also estimated as the bunch-weight-to-(bunch length)^2^ ratio, according to Tello and Ibáñez [34].

Five samples of 200 berries per treatment were randomly collected. From each sample, 150 berries were used in order to determine the berry weight, length and equatorial diameter (as described above), as well as average berry firmness (using a digital dynamometer SAUTER FH-M (Model FH 10, Sauter GmbH, Balingen, Germany), which was expressed as newton (N) force. Their berry and skin weights were also measured using a digital balance (Model TE64, Sartorius A.G., Goettingen, Germany) with a precision of 0.001 g, and the relative berry skin mass (%) was also calculated.

From the same samples, 50 berries per treatment were randomly collected, and berry skin color was evaluated using the color index (CIRG) for red grapes according to the CIELAB parameters L *(luminosity), h° (hue angle) and C * (chroma) [35], measured using Chroma Meter Miniscan XE Plus (Hunter Associates Laboratory, Inc., Reston, VA, USA).

### 2.5. Chemical and Organoleptic Analysis of Berries

The rest of the berries were randomly selected by creating five groups of 100 berries sampled from each treatment. Three groups were crushed, and the juice was filtered to obtain the must, from which total soluble solids (°Brix), titratable acidity (gL^−1^ tartaric acid) and the maturity index (°Brix/titratable acid) were determined, as described above. The fourth group of berries was frozen, and after few days, total berry skin anthocyanins were determined according to Ough and Amerine [36]. Anthocyanin concentrations were expressed as milligrams per gram of fresh berry weight.

Finally, the last group of berries was subjected to sensory evaluation. The sensory panel consisted of 25 participants who were representative of general consumers of table grapes. Each tasting session was carried out separately in a room with natural lighting. Each panelist consumed four berries from each treatment and used a 9-point hedonic scale to rate the flavor, aspect and crispness. The hedonic scale was the following: 1 = dislike extremely, 2 = dislike very much, 3 = dislike moderately, 4 = dislike slightly, 5 = neither like nor dislike, 6 = like slightly, 7 = like moderately, 8 = like very much and 9 = like extremely.

### 2.6. Statistical Analysis

Preharvest and harvest parameters were subjected to analysis of variance (ANOVA) using IMB SPSS Statistics 26.0 (IBM Corporation, Armonk, NY, USA). The significance of the differences between the mean values of each treatment was determined according to Duncan’s multiple range test at *p* < 0.05. Figures were illustrated using the SigmaPlot package, v.11 (Systat Software, San Jose, CA, USA).

## 3. Results and Discussion

### 3.1. Vineyard Microclimate

**Figure 1:**
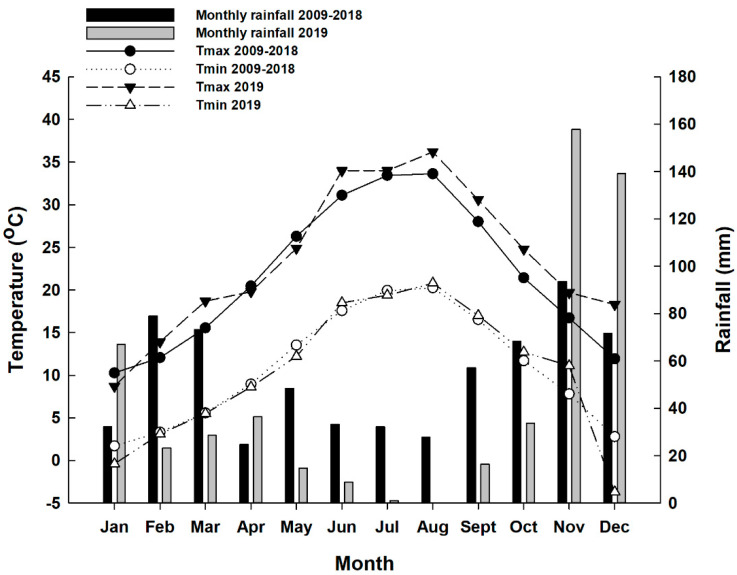
Seasonal trends (January–December) of monthly air temperature (T max, T min) and monthly rainfall recorded in 2019 and the last decade (2009–2018) near the trial site.

The microclimate conditions of the vineyard in the last decade and 2019, the year that the experiment was carried out, are reported in Figure 1. According to the data, every month in the last decade had some rainfall, but in 2019, almost all rainfall was recorded in January, November and December, which are the months of dormancy. Only 1 mm of rain was recorded in July and August 2019. The total rainfall (527 mm) was reduced by 18% compared to the 10-year average (641 mm). The mean air temperature in 2019 ranged from 0.62 to 16.6 °C and was higher than the mean air temperature of the decade. In almost all months of 2019, T max (0.6–6.7 °C) was higher than the T max of the decade, while a decrease in T min was recorded (−0.1 to −6.5 °C) compared to the last decade. In the months of grape maturation (from August to October), the mean temperatures were extremely high. In particular, August 2019 was warmer than usual (+2.6 °C). These data underline typical semi-arid Mediterranean conditions since the year 2019 was characterized by a rainless summer and a wetter winter compared to the last decade. Since temperature is one of the most important factors that affects fruit coloration, the high temperatures recorded in August, which is the month of grape maturity, could have a negative effect on the berry skin color and the biosynthesis of anthocyanins [9].

### 3.2. Evolution of Berry Weight and Berry Composition

**Figure 2:**
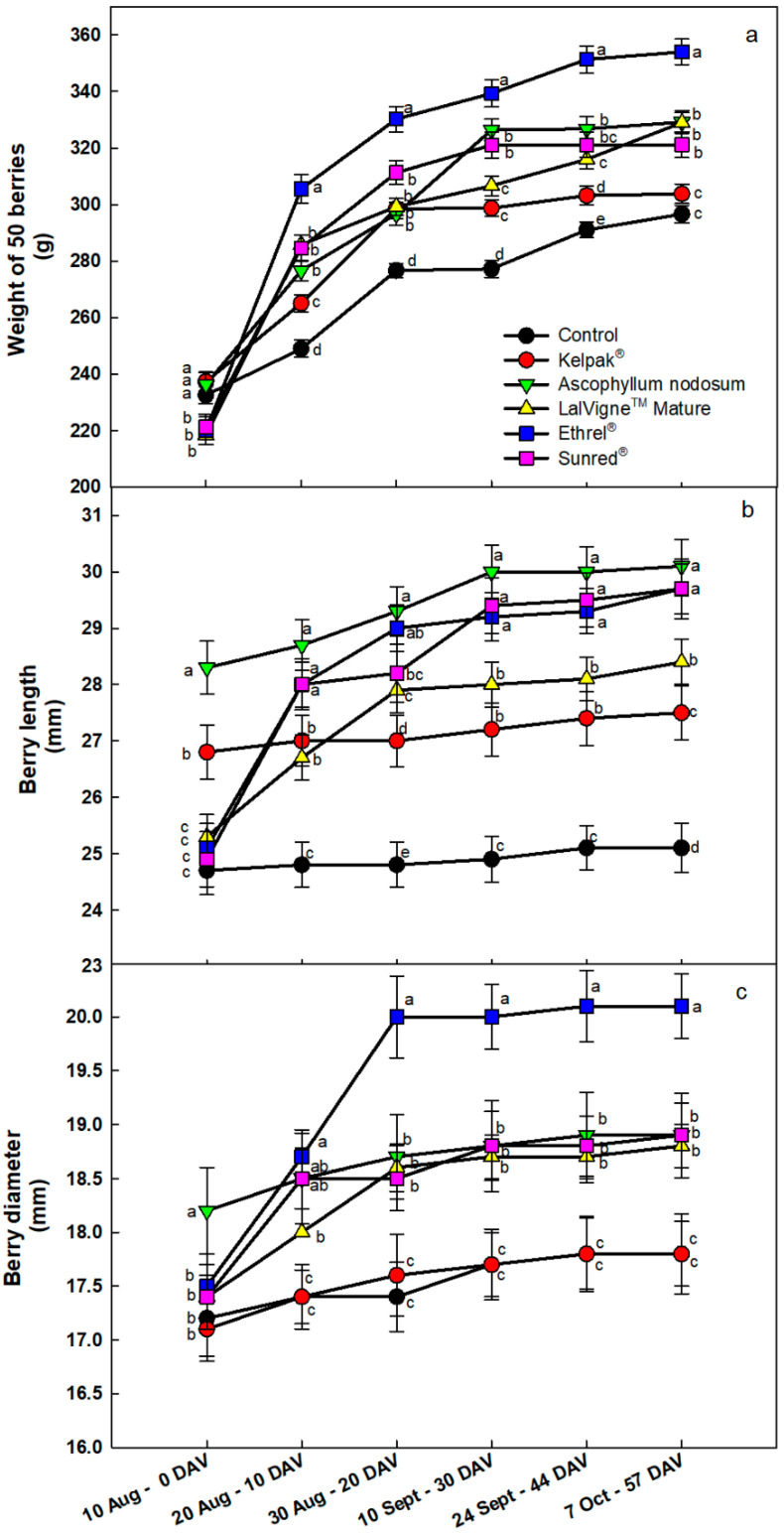
Evolution of the weight (panel **a**), length (panel **b**) and diameter (panel **c**) of 50 berries of table grape cv. Crimson Seedless from veraison (0 DAV) to harvest (57 DAV) after several preharvest canopy applications. The Control vines were untreated. Vertical bars indicate the standard errors of the means. Different letters indicate significant differences at *p* < 0.05 (Duncan’s multiple range tests). DAV = Days After Veraison.

The weights of 50 berries from the Kelpak^®^ and *Ascophyllum nodosum* treatments were significantly higher (+8%) than the control on the first sampling date, which was expected since these products were sprayed first (Figure 2a). The first sprays of the rest of the treatments had been applied by the second sampling date, and at this time, the berry weights of all treatments were higher compared to the control: +23% with Ethrel^®^ Top, +15% with Sunred^®^ and LM and +11% with AN compared to the control. On all subsequent sampling dates until harvest, the 50 sampled berries from the Ethrel^®^ Top treatment consistently had the highest weights, including at harvest (+19% vs. control), followed by the treatments with *Ascophyllum nodosum* and LalVigne™ Mature (+11% vs. control), Sunred^®^ (+8% vs. control) and Kelpak^®^ (+3% vs. control, Figure 2a).

The evolution of berry length and diameter is presented in Figure 2b,c, respectively. On the first sampling date, as expected, the berries from the AN and Kelpak^®^ treatments were longer: their length increases were +8.9% (26.8 mm) and +14.6% (28.3 mm) compared to the control, respectively. On the 10 August, at veraison, the control berries had almost reached their final lengths, and after this date, the length increase was only approximately +1.6% (0.4 mm, Figure 2b). The same observation was recorded for the Kelpak^®^ treatment, which produced an increase of +2.6% (0.7 mm, Figure 2b). However, the rest of the treatments (LalVigne™ Mature, Ethrel^®^ Top and Sunred^®^) caused an increase in berry length after their first spray (Figure 2b). On the final sampling date, longer berries were recorded for all treatments compared to the control. The *Ascophyllum nodosum* treatment led to longer berry lengths (+20% vs. control), followed by Sunred^®^ and Ethrel^®^ Top treatments, which had equivalent increases (+18% vs. control, Figure 2b). On the first sampling date, the *Ascophyllum nodosum* treatment had the greatest increase in berry diameter among all treatments (Figure 2c). However, the results on the last sampling date showed that the largest berry diameter was recorded for the Ethrel^®^ Top treatment (20.1 mm, +13% vs. control), followed by Sunred^®^, *Ascophyllum nodosum* and LalVigne™ Mature treatments, which had the same berry diameter of 18.9 mm (+6.2% vs. control). Kelpak^®^ berries had the same diameter as control berries (Figure 2c).

**Figure 3:**
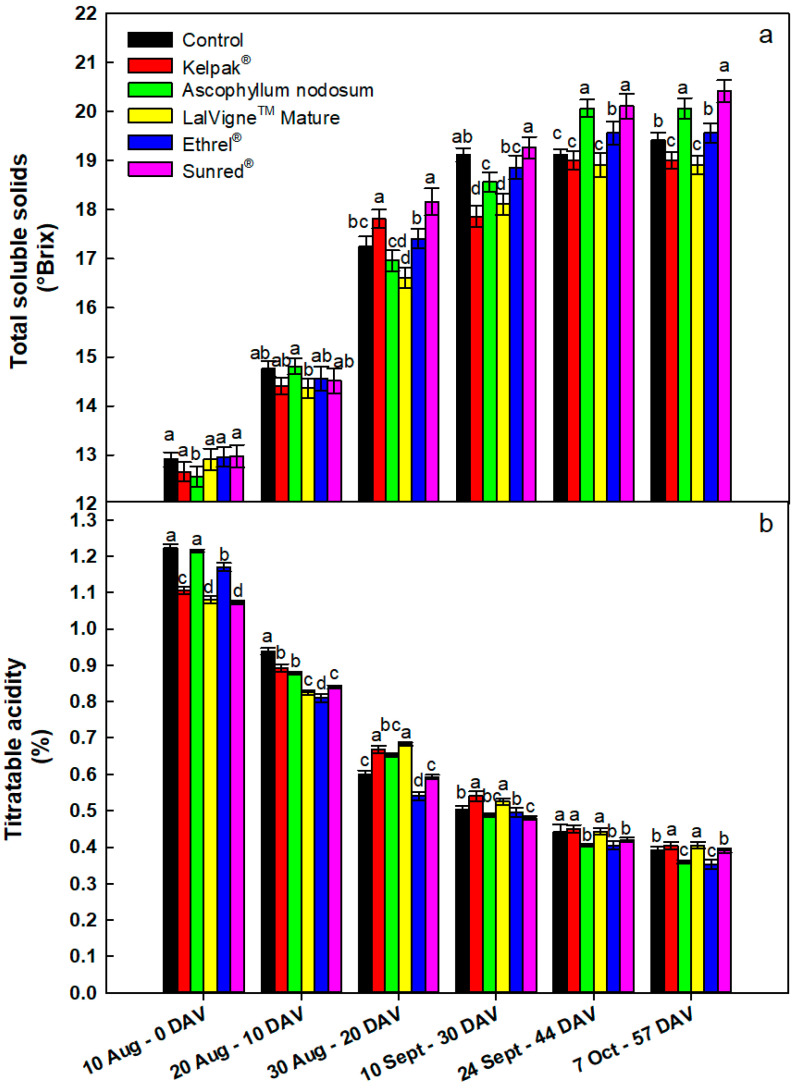
Evolution of total soluble solids (panel **a**) and titratable acidity (panel **b**) for table grape cv. Crimson Seedless after several preharvest canopy applications. The Control vines were untreated. Samples were taken from veraison (0 DAV) to harvest (57 DAV). Vertical bars indicate the standard errors of the means. Different letters indicate significant differences at *p* < 0.05 (Duncan’s multiple range tests). DAV = Days After Veraison.

On the first sampling date, the soluble solids concentration in the juice was the lowest in the *Ascophyllum nodosum* treatment compared to the other treatments (Figure 3a). On the second and third sampling dates, the sugar accumulation was the lowest in berries treated with LalVigne™ Mature compared to the other treatments. On the fourth sampling date (30 DAV), the musts from Kelpak^®^ and LalVigne™ Mature treatments had the lowest soluble solids concentrations compared to the other treatments. On the second-last sampling date, this trend was confirmed, and the values were equal to those of the control. On the last sampling date, the treatments with LalVigne™ Mature and Kelpak^®^ had significantly lower total soluble solids (−3% vs. control), while the Sunred^®^ treatment presented the highest (+5% vs. control). Between the 30th DAV and the last two sampling dates, the sugars only increased in the *Ascophyllum nodosum* treatment (+0.3° Brix), whereas the soluble solids concentration for the other treatments remained unchanged (Figure 3a). On the first sampling date (at veraison), titratable acidity was the lowest in LM and Sunred^®^ treatments, followed by Kelpak^®^ treatment, compared to the others (Figure 3b). After that and until harvest, the maximum decrease in titratable acidity was obtained in the Ethrel^®^ Top treatment: at harvest, Ethrel^®^ Top had the lowest TA (0.35%), followed closely by the AN treatment (0.36%), compared to the other treatments (Figure 3b). It is interesting to underline that almost all vines reached technological maturity before the harvest date (57 DAV), that is, before berries achieved the appropriate color (data not shown).

### 3.3. Productivity Parameters of Clusters and Berries at Harvest

Although the number of clusters per vine was not affected by treatments, vine productivity improved (Table 2). The Sunred^®^ treatment led to the highest yield at 14.8 kg/vine (+54% vs. control), followed by treatments with Ethrel^®^ Top and AN, which had yield increases of +18% and +14%, respectively, compared to the control. A significantly heavier cluster weight was obtained with the Sunred^®^ treatment (+68% vs. control), followed by Ethrel^®^ Top and *Ascophyllum nodosum* treatments, which had cluster weight increases of +29% and +25%, respectively, relative to the control.

Our data agree with previous studies that reported the promotion of berry growth after biostimulant applications [23,37,38]. Plant biostimulants contain diverse substances that can increase the photosynthetic capacity and chlorophyll content of leaves [39,40,41] and, therefore, may represent a potential tool for increasing yield. The same results obtained with Ethrel^®^ Top have been observed in previous experiments [42].

The application of *Ascophyllum nodosum* has been shown to increase the yield of numerous plants, such as apples of cv. ‘Fuji’ [43], pear trees [44] and strawberry plants [45], as well as vines of cv. ‘Flame Seedless’ [23] and cv. ‘Trakya Ilkeren’ [26]. Similar findings have been reported for ‘Flame Seedless’ [42], where Ethrel^®^ Top treatment increased the yield/vine due to increased cluster and berry weights.

In our experiment, the treatments did not affect the number of berries per cluster or the cluster dimensions. However, biostimulant applications did affect bunch compactness because of the larger berry dimensions, especially in the Sunred^®^ treatment, which resulted in more than double the cluster compactness (+101%) compared to the control (Table 2). The rest of the treatments had the same cluster compactness index, with increases ranging between 45% and 25% compared to the control. However, no differences in the incidence of bunch rot were observed between treatments (Table 2). Moreover, all the treatments that were sprayed at the veraison stage had higher berry firmness (Table 2). The highest firmness values were achieved by the Sunred^®^, LM and Ethrel^®^ Top treatments, which were 232%, 227% and 204% higher, respectively, than the control, while the rest of the treatments were not significantly different from the control (Table 2).

Differences in productivity between treatments were related to berry characteristics. The Ethrel^®^ Top and Sunred^®^ treatments had significantly higher berry weights (6.9 g), resulting in an increase of 49% compared to the control, followed by treatments with AN, LM and Kelpak^®^ (11%, 12% and 10% increases, respectively, as compared to the control, Table 3). The results of the Sunred^®^ treatment are in accordance with those of Deng et al. [41], who applied the treatment at a later stage of fruit ripening, while the AN results differ from those of Stino et al. [23] and Sabir et al. [39]. These results are related to the berry dimensions of these two treatments. Thus, Ethrel^®^ Top, Sunred^®^ and AN treatments produced berries with the same increases in length compared to the control. Moreover, the treatment with Ethrel^®^ Top had the largest berry diameter (20.13 mm), which was 13% larger than that of the control, followed by Sunred^®^, LM and AN treatments, which had the same berry diameter (~19.00 mm, Table 3). The berry skin mass was significantly higher relative to the control (10.9%) and highest among all treatments, followed by Kelpak^®^ and AN (10%), while no significant differences were observed among the other treatments (Table 3). The observed changes in berry characteristics were only reflected in juice volume/100 berries for the AN and Ethrel^®^ Top treatments (+78% and +71%, respectively, vs. control), and the control had the lowest juice volume (Table 3). Similar responses to AN application have been previously reported [23,39].

In the present study, the greatest increase in yield was obtained in the Sunred^®^ treatment, followed by Ethrel^®^ Top. These yield increases were the result of increased berry and cluster weights. One of raw materials of the Sunred^®^ product is seaweed extract, which contains phytohormones such as abscisic acid, gibberellic acid, cytokinins, indole acetic acid and polyamines. The direct effects of these substances include the stimulation of cell division and cell enlargement, resulting in increased fruit size, as previously reported [46,47].

### 3.4. Quality at Harvest

Significant differences between treatments were also found for total soluble solids, titratable acidity, relative soluble solids/acidity and anthocyanin content of berries. The highest TSS value (20.9 Brix, +6% vs. control) was observed in the treatment with Sunred^®^ (Table 4). On the other hand, the TSS values in Kelpak^®^ and LM treatments were lower (18.91 and 18.88 Brix) than those of the control (19.71 Brix). The effects of Sunred^®^ are probably linked to increased primary metabolism in the vines after the application of this product since it is able to increase the chlorophyll content [48] and photosynthetic activity of the leaves [41]. Moreover, Sunred^®^ contains potassium; according to Römheld and Kirby [49], potassium has an important role in photosynthesis, carbohydrate metabolism and the transportation of nutrients from the leaves to the fruits. According to Khan et al. [24], the increase in the total soluble solids in the juice is possibly linked to specific enzymes in seaweed extracts that promote the synthesis of several proteins, phytohormones, amino acids and sugars.

Titratable acidity was affected by the treatments. The Ethrel^®^ Top treatment resulted in the lowest titratable acidity, which was 14% lower than the control value (Table 4). This result agrees with those of Gallegos et al. [50] and Kassem et al. [42], who reported that treatment of *cv.* ‘Tempranillo’ and Flame ‘Seedless’ with Ethrel^®^ Top decreased the total acidity of the juice. This occurs because Ethrel^®^ Top induces an increase in mitochondrial oxidation of malic acid [51,52]. The results of the Sunred^®^ treatment in this study were similar in *cv.* ‘Red Globe’ in the study by Deng et al. [41].

The highest maturity index was obtained by Ethrel^®^ Top (+14% vs. control), followed by AN and Sunred^®^ (+13% and 12% vs. control, respectively), while the Kelpak^®^ treatment had the lowest maturity index, which was the same as the value of the control (Table 4). Our results are in accordance with the results of other studies for Sunred^®^ [41], *Ascophyllum nodosum* [23,24] and Ethrel^®^ Top [43].

The anthocyanin content of berries from the Sunred^®^ treatment was significantly higher than that obtained in all other treatments and the control (+21% vs. control, Table 4). A similar result for total anthocyanins in berries was reported for ‘Red Globe’ grapes after Sunred^®^ application [41]. Seaweed extracts are the main ingredient of Sunred^®^, and these extracts have been proven to increase leaf chlorophyll content and plant photosynthetic activity [40,41]. More efficient photosynthesis translates to a greater production of monosaccharides that are able to react with anthocyanidins to form anthocyanins [52]. Moreover, Sunred^®^ has been reported to increase the expression of genes that are involved in anthocyanin biosynthesis, by probably stimulating the promoters of these genes. Sunred^®^ is rich in oxylipins, phenylalanine and monosaccharides, which are potential initiators of anthocyanin biosynthesis. Indeed, anthocyanins are synthesized in the general phenylpropanoid pathway [53], while oxylipins act as precursors of many cyclopropanium compounds, which are involved in many processes that are connected to maturation, chlorophyll deconstruction, anthocyanin synthesis and the concentration of phenolic compounds [54]. Potassium is an important mineral for enzyme activation, photosynthesis and osmotic regulation in grapes [55], as well as for their quality and yield [56,57]. Since Sunred^®^ contains inorganic potassium, it may play a role in increasing the activity of enzymes that are involved in anthocyanin biosynthesis in the skin of berries.

### 3.5. Berry Color Characteristics and Sensory Attributes

**Figure 4:**
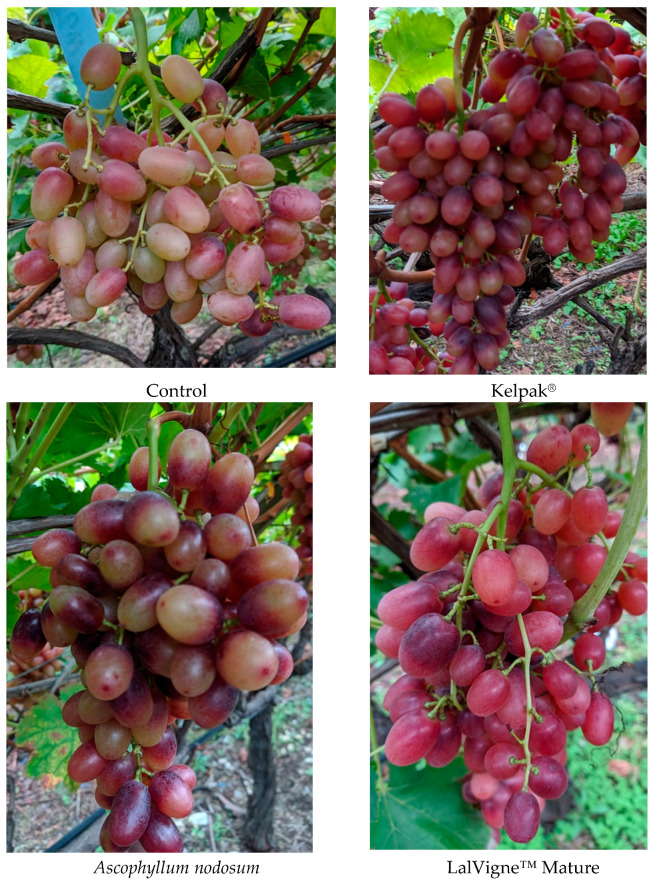

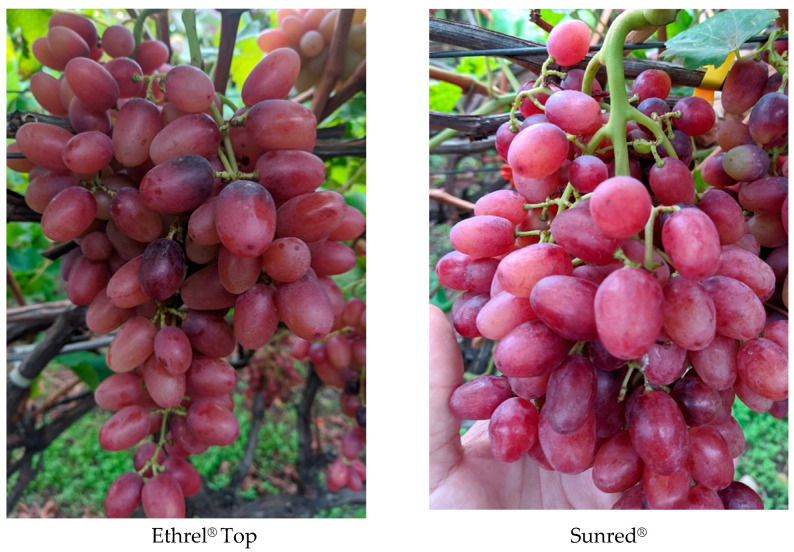
Clusters at harvest of table grape cv. Crimson Seedless after several preharvest canopy applications and compared to cluster from untreated vines (Control).

Higher α* values and lower b* values were detected in all treatments, and consequently, the berries in these groups had higher saturation (C*) values (6.42 and 6.62), except for the Kelpak^®^ treatment, and lower hue angles (*h°*) than the control berries (Table 5). The highest a* and lowest b* values, which were observed in the treatments with Sunred^®^ and Ethrel^®^ Top, could indicate that their berry color was purer. Although there were no significant differences in CIRG among different treatments and the control, the value with Sunred^®^ (7.25) was larger than that of the control (6.86). Similar results after treatment with Sunred^®^ were reported for ‘Red Globe’ grapes [41,58]. In fact, when CIRG values are higher than 4.6, the color of the berries is considered violet, and when the values are higher than 5.3, the color is considered dark violet [35]; these findings are illustrated by photos of the clusters at harvest (Figure 4).

In the sensory evaluation results, Sunred^®^ was the only treatment that received higher ratings for all four sensory characteristics (flavor, aspect, crispness and overall acceptability). The second most preferred berries in the consumer panel were derived from the Ethrel^®^ Top treatment (Table 5).

## 4. Conclusions

In recent years, red table grape cultivars growing in semi-arid Mediterranean conditions in Greece have been increasingly unable to achieve their desirable color, so plant growth regulators have been widely used by growers to obtain the best fruit quality without reducing yield. However, few studies have compared ethephon, which is extensively used to promote berry color and improve fruit quality, with biostimulants or other alternative substances that may be able to replace it.

To our knowledge, this work is the first attempt to simultaneously study the effects of these substances on the fruit yield and quality of table grapes *cv.* Crimson Seedless at harvest. The greatest improvements in the red berry color were achieved with Sunred^®^ (at a dose of 4 L ha^−1^) and Ethrel^®^ Top (250 ppm plus glycerol at 1%), each applied at veraison and 10 days later. Moreover, the Sunred^®^ treatment improved the overall acceptability perceived by consumers.

Since preharvest applications of the Sunred^®^ biostimulant may constitute not only a viable alternative to ethephon but also a powerful and sustainable tool for the organic production of table grapes with premium quality, further studies are needed to elucidate the mechanisms of actions implicated after biostimulant applications.

## Figures and Tables

**Table 1:** Trade names, chemical composition, phenological stages, dates of applications and doses of the products used in the experiment. V: Veraison; DAV: Days After Veraison.

Trade Names	Chemical Composition	Phenological Stages *, Dates and Doses of the Applications					
		1st Spray	2nd Spray	3rd Spray	4th Spray	5th Spray	Doses
Kelpak^®^	Seaweed extract of *Ecklonia maxima* containing macro- and micronutrients, amino acids, vitamins, plant hormones and carbohydrates	E − L = 15 (8 leaves separated, shoot elongating rapidly; single flowers in compact groups, 25/05)	E − L = 12 (full bloom; 50% caps off, 03/06)	E − L = 31 (berries pea-size, 13/06)	−	−	3 L Ha^−1^
Cytolan^®^ Concentrated Powder (*Ascophyllum nodosum*)	Solid seaweed extract of *Ascophyllum nodosum,* 1% total N, K_2_O 10%, 600 ppm plant hormones, 35% carbohydrates, 3% alginic acid, 9% mannitol and 45–55% organic matter; 2.8% Total Nitrogen (N) 17% Water-soluble Potassium Oxide (K_2_O); 16% Alginic acid; 4.3% Mannitol	E − L = 15 (8 leaves separated, shoot elongating rapidly; single flowers in compact groups, 25/05)	E − L = 12 (full bloom; 50% caps off, 03/06)	E − L = 31 berries pea-size, 13/06)	E − L = 33 (Berries still hard and green, 18/06)	E − L = 34 (Berries begin to soften; Brix starts increasing, 22/06)	4 g L^−1^ Vine^−1^ (according to Stino et al. [23])
LalVigne™ Mature	100% natural, inactivated wine yeast (*Saccharomyces cerevisiae*) derivatives	E − L = 35 (Veraison, 10/08)	E − L = 36 (Berries with intermediate Brix values, 10 days after veraison (10 DAV), 20/08)				1.5 kg ha^−1^
Ethrel^®^ Top	40% ethephon (2-chloroethyl phosphonic acid)	E − L = 35 (Veraison, 10/08)	E − L = 36 (Berries with intermediate Brix values, 10 days after veraison (10 DAV), 20/08)				250 ppm plus glycerol at 1% (according to Farag et al. [33])
Sunred^®^	26.6 g/L organic N, 13.3 g/L of mineral N, 93.1 g/L of K_2_O, 186.2 g/L of organic C, oxylipins, phenylalanine, methionine, monosaccharides, glucose, amino acids, sodium hydroxide and citric acid (per liter)	E − L = 35 (Veraison, 10/08)	E − L = 36 (Berries with intermediate Brix values, 10 days after veraison (10 DAV), 20/08)				4 L ha^−1^

* According to the modified E-L system [32].

**Table 2:** Harvest data, cluster morphology and bunch rot incidence in grapevines cv. Crimson Seedless after several preharvest canopy applications, in comparison with untreated vines (Control).

Treatment	Yield (kg/vine)	Clusters Per Vine (*n*°)	Cluster Weight (g)	Berries Per Cluster (n°)	Cluster Length (cm)	Cluster Width (cm)	Cluster Compactness Index (g/(cm)^2^)	Berry Firmness (N)	Bunch Rot Incidence (%)
Control	9.64 d	20.8 a	463.60 d	97.6 a	22.8 a	17.1 a	20.33 c	1.66 b	0.4 a
Kelpak^®^	10.09 c	19.0 a	531.22 c	100.8 a	21.7 a	18.3 a	24.48 bc	2.65 b	0.8 a
*Ascophyllum nodosum*	11.03 b	19.0 a	580.39 bc	91.4 a	21.3 a	18.0 a	27.25 b	2.44 b	2.0 a
LalVigne^TM^ Mature	10.66 bc	19.5 a	546.42 c	96.2 a	21.3 a	17.3 a	25.65 b	5.42 a	1.0 a
Ethrel^®^	11.40 b	19.0 a	634.34 b	97.2 a	20.8 a	17.0 a	28.84 b	5.05 a	0.0 a
Sunred^®^	14.82 a	19.0 a	779.95 a	113.2 a	19.6 a	17.1 a	40.81 a	5.51 a	1.2 a

Mean values followed by different letters within the same column indicate significant differences according to Duncan’s multiple range test (*p* < 0.05).

**Table 3:** Berry characteristics at harvest in grapevines cv. Crimson Seedless after several preharvest canopy applications, in comparison with untreated vines (Control).

Treatment	Berry Weight (g)	Berry Length (cm)	Berry Diameter (cm)	Berry Skin Mass (%)	Juice Volume of 100 Berries (mL)
Control	4.75 e	25.16 c	17.85 c	12.11 a	102.7 d
Kelpak^®^	5.27 d	25.58 b	17.84 c	10.91 b	113.3 cd
*Ascophyllum nodosum*	6.35 b	30.15 a	18.94 b	10.61 b	182.3 a
LalVigne^TM^ Mature	5.68 c	28.42 b	18.80 b	9.67 c	153.3 b
Ethrel^®^	6.88 a	30.67 a	20.13 a	9.80 c	176.0 a
Sunred^®^	6.89 a	30.03 a	18.85 b	8.68 c	120.0 c

Mean values followed by different letters within the same column indicate significant differences according to Duncan’s multiple range test (*p* < 0.05).

**Table 4:** Quality of fruit at harvest in grapevines cv. Crimson Seedless after several preharvest canopy applications, in comparison with untreated vines (Control).

Treatment	Total Soluble Solids (TSS, °Brix, %)	Titratable Acidity (TA, %)	Maturity Index (TSS/TA)	Total Anthocyanins (mg/g Fresh Berry Weight)
Control	19.71 bc	0.398 a	49.59 b	0.335 b
Kelpak^®^	18.91 c	0.383 b	49.44 b	0.291 b
*Ascophyllum nodosum*	20.38 ab	0.363 c	56.22 a	0.340 b
LalVigne^TM^ Mature	18.88 c	0.378 b	50.01 b	0.297 b
Ethrel^®^	19.73 bc	0.348 d	56.78 a	0.323 b
Sunred^®^	20.94 a	0.378 b	55.47 a	0.407 a

Mean values followed by different letters within the same column indicate significant differences according to Duncan’s multiple range test (*p* < 0.05).

**Table 5:** The color characteristics and sensory attributes of berries at harvest in grapevines cv. Crimson Seedless after several preharvest canopy applications, in comparison with untreated vines (Control).

Treatment	α*	b*	L*	C*	*h°*	CIRG	Flavor	Aspect	Crispness	Overall Acceptability
Control	6.2 c	9.6 a	19.9 a	15.9 bc	57.6 a	6.86 a	6.75 a	4.83 c	7.83 a	6.5 b
Kelpak^®^	8.5 bc	6.4 b	19.9 a	14.8 c	37.4 b	6.93 a	6.42 ab	5.46 bc	7.00 a	6.1 bc
*Ascophyllum nodosum*	11.5 ab	8.4 ab	23.4 a	19.9 ab	37.2 b	5.52 a	6.83 a	6.00 b	7.25 a	6.7 b
LalVigne^TM^ Mature	10.9 ab	6.9 ab	19.1 a	17.8 ab	33.1 b	6.70 a	5.58 b	5.92 b	5.33 b	5.6 c
Ethrel^®^	12.3 a	8.5 ab	21.0 a	20.7 a	33.2 b	6.36 a	6.92 a	7.92 a	6.92 a	7.2 ab
Sunred^®^	11.6 ab	5.9 b	18.0 a	17.5 ab	25.4 b	7.25 a	7.08 a	8.03 a	7.75 a	7.6 a

Mean values followed by different letters within the same column indicate significant differences according to Duncan’s multiple range test (*p* < 0.05).

## Data Availability

Data are contained within the article.

## Abbreviations

Minimum temperature	T min
Maximum temperature	T max
Ascophyllum nodosum	AN
LalVigne™ Mature	LalVigne™ Mature
Days after veraison	DAV
Total soluble solids	TSS
Titratable acidity	TA
